# Impact of Methicillin-Resistant *Staphylococcus aureus* on Treatment of Hand Infections

**Published:** 2009-11-20

**Authors:** Scott D. Lifchez, David A. Harriman

**Affiliations:** ^a^Department of Surgery, Division of Plastic Surgery, Johns Hopkins University, Baltimore, Md; ^b^Department of Emergency Medicine, Johns Hopkins University, Baltimore, Md

## Abstract

**Objective:** With the increasing incidence of methicillin-resistant *Staphylococcus aureus* (MRSA) in hand infections in urban centers, multiple studies have recommended using MRSA-effective antibiotics as first-line treatment of hand infections. This study assesses the effect of adopting this recommendation for the treatment of hand infections at the authors' hospital. **Methods:** Patients with hand infections drained in the authors' hospital were prospectively enrolled in an observational study over a 12-month period. Culture results and response to treatment were recorded. **Results:** Twenty-two patients met inclusion criteria. Eleven of 14 patients with *S aureus* infections had MRSA. All of these patients responded to the initial antibiotic selected. Two patients had infections that did not respond to trimethoprim-sulfamethoxazole. One grew group A *Streptococcus* infection, and the other had lymphangitic streaking that suggested *Streptococcus* infection. **Conclusion:** Because of the high prevalence of MRSA among hand infections at the authors' institution, we continue to prescribe MRSA-effective antibiotics as first-line treatment of hand abscesses. Close follow-up is still necessary to confirm that each patient has responded appropriately to treatment or to allow modification of the treatment plan if the patient has not responded to treatment.

Since the early reports of methicillin resistance in *Staphylococcus aureus*,^1^,^2^ there has been great interest in monitoring its prevalence as an infecting organism. Multiple recent reports have documented the increase in frequency of methicillin resistance among *S aureus* taken from abscesses and other infections.^3^,^4^ Several reports in the past few years have specifically documented the rise of methicillin-resistant *S aureus* (MRSA) in community-acquired infections. Mechanisms of spread of methicillin resistance have also been studied.^5^,^6^

Multiple recent reports have specifically focused on the increase of MRSA in hand infections.^7^–^11^ These reports came out of major urban hospitals in Chicago and Dallas, where the prevalence of MRSA in the community is high. These studies concluded that in communities where MRSA prevalence is high, strong consideration should be given to choosing an antibiotic effective against MRSA as first-line treatment of abscesses of the hand after appropriate drainage.

The urban area surrounding the authors' hospital also has a high MRSA prevalence rate. On the basis of this and the above reports, our ER and hand services adopted the recommendations of the studies^7^,^9^ and began prescribing antibiotics effective against MRSA as first-line treatment for discharge after drainage of a hand infection in patients with no known increased risk (such as diabetes mellitus and intravenous [IV] drug abuse) for gram-negative infection. Based on low-cost and easier twice-a-day dosing, trimethoprim-sulfamethoxazole (TMP-SMX) was selected as the preferred antibiotic, as it was in the above studies.

The purpose of this study was to prospectively monitor the results of treatment of hand infections at the authors' institution after adoption of TMP-SMX as first-line treatment of patients discharged from the ER after drainage of a hand abscess.

## METHODS

Patients were collected prospectively from October 2007 to September 2008. All patients with inflammation at or distal to the radiocarpal joint presenting to the Johns Hopkins Bayview Medical Center emergency department were evaluated to determine whether they were candidates for the study. Patients were drained in the emergency department or operating room as deemed appropriate by the treating surgeon. Patients with no identifiable fluid collection to drain were admitted for IV antibiotics or discharged on oral antibiotics depending on severity of inflammation and medical comorbidities. Patients who received more than 24 hours of antibiotics (greater than 24 hours between initiation of antibiotic therapy and identification of a drainable fluid collection) were excluded from the study. All patients were managed with the standard treatment for hand infections. There was no alteration in patient care practices due to study participation.

All fluid collections drained were sent for culture and sensitivities. Patients were given systemic antistaphylococcal antibiotics orally (if discharged from the ER) or intravenously (if admitted to the hospital). Because of the high frequency of MRSA in our hospital and based on multiple previous studies (see the introductory paragraphs), IV vancomycin and oral TMP-SMX double strength were used for all patients unless there was a known allergy. Patients with allergies received clindamycin. Patients felt to be at increased risk of gram-negative infection (diabetics, IV drug abusers, or history of cat or dog bite) received broadened-spectrum antibiotic coverage (IV ampicillin/sulbactam or oral amoxicillin/clavulonic acid, or a quinolone if penicillin allergic) in addition to coverage for MRSA.

Patients were followed with serial examination. Patients admitted to the hospital were reassessed at least once a day. Those sent home from the ER were instructed to follow-up 2 days later. Patients discharged from the ER who did not follow up within 72 hours would have been excluded from the study, but this situation did not occur in our study population.

All patients received antibiotics for 7 to 10 days and local wound care to the drainage site until resolution of inflammation. If culture and sensitivity information indicated, antibiotic coverage was changed once this information became available.

This study was approved by the Johns Hopkins Medical Institutions investigational review board (IRB file number: NA_00011969).

## RESULTS

Twenty-two patients met inclusion criteria. As with previous studies, our study showed a high prevalence of MRSA. Eleven of 14 *Staphylococcus* cultures were methicillin resistant. Sensitivities of *Staphylococcus* are shown in Table 1. All MRSA cultures were sensitive to TMP-SMX, but sensitivity to other antibiotics was highly variable, ranging from 27% to 92%.

One patient's cultures grew out *Pseudomonas* species. This patient had a history of IV drug abuse. One patient, with a recent history of cat bite, grew *Pasteurella* from her cultures. Both of these gram-negative rods were sensitive to quinolones, aminoglycosides, and anti-pseudomonnal penicillins and cephalosporins. Both patients had significant inflammation at the time of presentation and were admitted to the hospital after drainage. Both patients received IV vancomycin and piperacillin-tazobactam until sensitivities were available. Both patients were discharged on oral levofloxacin. They were followed up in clinic and their wounds and inflammation went on to resolve without further intervention.

We also identified one patient who grew out group A *Streptococcus* species from their cultures. The patient did not demonstrate lymphangitic streaking during the initial ER visit. The patient had been drained in the emergency department and discharged. Follow-up in the office 2 days after drainage (coinciding with when culture and sensitivity results became available) demonstrated persistent inflammation (minimally improved from prior to drainage) but no residual purulence. The patient had been discharged on oral TMP-SMX DS per our standard treatment protocol. Antibiotics for this patient were changed to amoxicillin-clavulonic acid. When this patient was seen in follow-up an additional 2 days later, a marked improvement of the inflammation was noted. A second patient had a similar clinical history to the above patient, but his culture ultimately came back with no growth. He had been discharged from the ER on oral TMP-SMX DS. At the time of the clinic visit, there was persistent inflammation and questionable lymphangitic streaking, but no residual fluctuance to allow for a new culture to be obtained. Based on this information and our experience with the previous patient who grew out group A *Streptococcus*, we changed antibiotics to amoxicillin/clavulonic acid. Two days later, the inflammation and lymphangitic streaking were markedly improved, and inflammation was resolved an additional week later. We suspected that this patient, despite the negative culture, may also have had TMP-SMX-resistant *Streptococcus*. For both of these patients, the drainage incisions went on to heal with no residual inflammation by the next follow-up visit 1 week later.

## DISCUSSION

Multiple previous studies have documented the increasing frequency of MRSA cultures for infections of the hand and other locations.^3^,^4^,^7^–^9^ Reasons for this increase have been proposed.^5^,^6^ Many of these studies concluded that antibiotics effective against MRSA should be selected as first-line treatment of patients with infections in which *S aureus* is considered likely to be a pathogen.

The authors' hospital antibiogram shows a high prevalence of MRSA. For this reason, the recommendations of the above studies were adopted. Anti-MRSA medications were selected as primary treatment of community-acquired hand infections. This study reviews our experience with the treatment of hand infections since the adoption of this policy. While patients with significant comorbidities and those admitted to the hospital were covered with broad spectrum antimicrobials, those patients discharged from the ER were routinely prescribed TMP-SMX DS alone. The 2 patients, whose cultures grew gram-negative rods (one *Pseudomonas*, one *Pasteurella*), were admitted because of the severity of inflammation surrounding the abscess as well as for significant comorbidities that raised our suspicion for gram-negative infection (one was an IV drug abuser and the other presented 2 days after a cat bite). As such, they were covered from the time of admission with antimicrobials effective against the pathogen in their abscesses. The one patient with *Streptococcus* and the second patient with suspected *Streptococcus* had been sent home on TMP-SMX as described above. They ultimately required treatment with amoxicillin-clavulonic acid to resolve inflammation. Although these 2 patients had a slight delay in resolution of their symptoms due to selection of an anti-MRSA medication that was not effective against the *Streptococcus* that ultimately grew out in culture for one and was suspected on the basis of the clinical course of the other, given the continued high prevalence of MRSA in our community, our practice has continued to recommend MRSA-effective antibiotics as initial treatment of patient presenting with community-acquired hand infections.

For communities where MRSA prevalence is high, TMP-SMX remains a good empiric choice for hand infections. For those patients who do not respond to TMP-SMX or who show findings suggestive of *Streptococcus* infections, additional coverage with a better antistreptococcal medication such as amoxicillin-clavulonic acid is a reasonable option. A first-generation cephalosporin such as cephalexin is also a reasonable alternative but may increase risk of noncompliance due to 4 times per day dosing.

MRSA samples in our patients were also highly sensitive to clindamycin (82%) and tetracycline (92%). Clindamycin may be less likely to fail patients with *Streptococcus* infection but does carry a higher risk of antibiotic-associated diarrhea, higher cost, and higher risk of noncompliance due to QID dosing. Tetracycline family medicines such as doxycycline could be used as well, but it also carries a risk failure in *Streptococcus* infections as well as a risk of allergy.

For any hospital setting, the choice of antimicrobial treatment of patients who undergo drainage of a hand infection must be based on a number of factors: hospital/community prevalence of MRSA, patient comorbidities, and ability/likelihood of the patient to comply with antibiotic regimens requiring multiple doses per day.

Our study is limited by its small patient number. However, on the basis of our results, we continue to treat hand infections with drainable abscesses with vancomycin (if admitted) or TMP-SMX DS (if discharged from the ER) as primary therapy. Failures of this treatment algorithm continue to be uncommon.

The authors have no financial interests in any of the material discussed in this manuscript.

## Figures and Tables

**Table 1:** Specimens (%) sensitive to the antibiotics^*a*^

	Oxacillin	Clindamycin	Erythromycin	Ciprofloxacin	Tetracycline	Trimethoprim-sulfamethoxazole
MRSA^*b*^	0	82	27	0	92	100
MSSA^*c*^	100	100	100	100	100	100

^*a*^MRSA indicates methicillin-resistant *Staphylococcus aureus*.

^*b*^There were 11 specimens in this group.

^*c*^There were 3 specimens in this group.
